# Obesity- attributable costs of absenteeism among working adults in Portugal

**DOI:** 10.1186/s12889-022-13337-z

**Published:** 2022-05-15

**Authors:** Kelli Destri, Joana Alves, Maria João Gregório, Sara Simões Dias, Ana Rita Henriques, Nuno Mendonça, Helena Canhão, Ana Maria Rodrigues

**Affiliations:** 1 Comprehensive Health Research Centre, NOVA Medical School, Universidade Nova de Lisboa, 1169-056 Lisboa, Portugal; 2 EpiDoC Unit, CEDOC, NOVA Medical School, Universidade Nova de Lisboa, Lisboa, Portugal; 3 Comprehensive Health Research Center (CHRC), NOVA National School of Public Health, Public Health Research Centre, Universidade NOVA de Lisboa, Lisbon, Portugal; 4 Programa Nacional Para a Promoção da Alimentação Saudável, Direção-Geral da Saúde, Lisbon, Portugal; 5 Faculdade de Ciências da Nutrição E Alimentação, Universidade Do Porto, Porto, Portugal; 6 Center for Innovative Care and Health Technology, CiTechCare, Instituto Politécnico de Leiria/Escola Superior de Saúde, Leiria, Portugal; 7 National School of Public Health, UNL, Lisboa, Portugal; 8 Hospital Dos Lusíadas, Lisboa, Portugal

**Keywords:** Obesity, Absenteeism, Costs, Cohort Studies

## Abstract

**Background:**

Obesity leads to poor health outcomes and may adversely affect work productivity. This study, aimed to investigate the obesity- attributable costs of absenteeism among working adults in Portugal.

**Methods:**

The study population included individuals actively working at baseline from the Epidemiology of Chronic Diseases Cohort (EpiDoC), a large Portuguese population-based prospective study. Body mass index was measured at baseline and in two follow-up interviews. Absenteeism in each wave of the EpiDoC was assessed by the question “Did you have a sick leave in the previous 12 months? yes/no”, followed by “How many days did you miss work due to sickness in the previous twelve months?”. Body mass index (BMI) was classified into underweight, normal weight, overweight, and obese, based on the standard World Health Organization definition.

Association between obesity and absenteeism was estimated with the negative binomial regression model adjusted for BMI, chronic diseases, and lifestyle. Obesity- attributable costs were calculated using lost gross income during the time absent from work, through the human-capital approach.

**Results:**

The EpiDoC included 4338 working adults at baseline. Of these, 15.2% were obese at the beginning of the study and 22.7% of the population had been absent from work in the last 12 months. Participants with obesity missed 66% more days at work (IRR: 1.66; CI 95%:1.13–2.44; (*p* = 0.009.) than those with normal weight. The odds of having been absent from work were 1.4 times higher in obese compared to non-obese individuals (CI 95%: 1.18–1.67; *p* < 0.01) adjusted to sex and type of work. Obese individuals missed 3.8 more days per year than those with normal weight (95%CI: 3.1–4.5). Extrapolating to the entire Portuguese working population, absenteeism due to obesity incurred an additional cost of €238 million per year.

**Conclusion:**

Obesity imposes a financial burden due to absenteeism in Portugal. Employers and national health regulators should seek effective ways to reduce these costs.

**Supplementary Information:**

The online version contains supplementary material available at 10.1186/s12889-022-13337-z.

## Background

Obesity, A condition resulting from excess accumulation of body fat, is a major public health problem responsible for increased patient morbidity and mortality [1, 2]. It is estimated that by 2030 about 38% of the world's adult population will be obese [3]. In Europe, 53.1% of the population were either overweight or obese [4]. In Portugal, according to the latest studies of the National Health Examination Survey (INSEF), the prevalence of obesity has almost doubled from 2003 to 2015 (14.2% vs 28.6) [5]. The burden of obesity is a major problem for societies, and obesity has important consequences for health and the economy [6, 7]. The impact of obesity on the economy can be quantified by direct or indirect costs. Direct costs are defined as the costs involved in treating obesity and obesity-related chronic conditions [8–10]. In economic evaluations, indirect costs usually represent the loss of production due to decreased health or disease. Thus, the indirect costs components are absence from work (absenteeism), reduced productivity (presenteeism) while at work, and reduced informal work (e.g., daily activities at home or leisure) [11]. This study only considers the latter, i.e., the cost of absenteeism from work which is usually valued through gross wages (human capital approach).The impact of the indirect costs of obesity in some regions of Europe, such as Germany, can range from 0.47% to 1.21% of gross domestic product (GDP) [12–14]. while in Italy, the total attributable costs obesity amount to €13.34 billion in 2020, with indirect costs of €5.45 billion, of which €2.62 billion were due to absenteeism [15]. Globally the economic impact of obesity was estimated to be $2 trillion or 2.8% of annual global GDP [16]. However, few studies have measured obesity- attributable costs of absenteeism in Europe, and almost all existing studies were carried out in northern and central Europe. Thus, the total costs associated with obesity in Europe, particularly in southern Europe, are still poorly explored.

To fill in this knowledge gap, in this work, we will use the EpiDoC cohort to estimate the obesity-attributable costs of absenteeism among working adults in Portugal.

## Methods

### Study population

The data analyzed in this study were collected as part of the Epidemiology of Chronic Diseases Cohort (EpiDoC), initiated in 2011. EpiDoC is a closed prospective cohort that aimed to create a large population database for medical and health-related research in Portugal. It comprises a representative sample of adults (≥ 18 years old) who were non-institutionalized and living in private households in mainland Portugal or its islands (Azores and Madeira). Participants were selected using multistage, random sampling, as described elsewhere [17], and baseline assessment involved a face-to-face interview. All participants enrolled in EpiDoC 1 (2011–2013; *n* = 10,661) and those who provided their telephone number were enrolled in the subsequent follow-up evaluations, EpiDoC 2 (2013–2015) and EpiDoC 3 (2015–2016) [18]. Data were collected via a structured questionnaire through phone interviews, using a computer-assisted personal interview system.

**Fig. 1:**
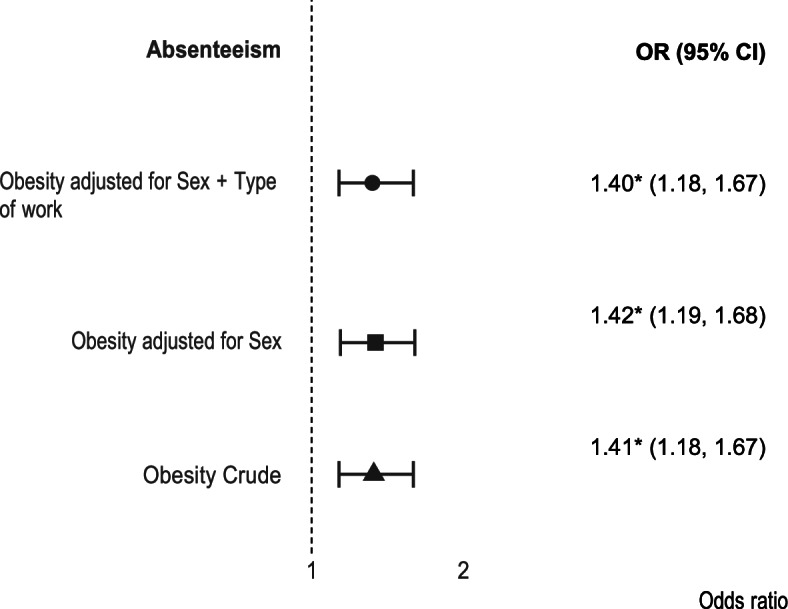
Flowchart describing the population eligible of the study

The population of interest for the present study included all subjects ≥ 18 years of age who were active workers (employed full-time or part-time) at baseline and subsequent waves and were participants of the national representative cohort of Portuguese adults (EpiDoC cohort). A flowchart illustrating our study population in each of the three evaluation periods is presented in Fig. 1.

### Absenteeism

Absenteeism was assessed based on the following questions: 1)"Have you had a sick leave in the last 12 months? yes/no"; 2) " How many days did you miss work due to being sick in the last 12 months?". These questions were asked at baseline and in the two follow-up waves. In the follow-up the question was slightly different—“How many days did you miss work due to hospital admissions, consultations, sick leave in the last 12 months?” The absenteeism variable was considered in 2 different ways: binary (absent from work: yes/ no) and continuous (number of days absent from work).

### BMI

Self-reported weight and height were used to calculate BMI (weight/height^2^, in kg/m^2^). BMI was categorized into: underweight, < 18.5 kg/m^2^; normal weight, 18.5–24.9 kg/m^2^; overweight, 25–29.9 kg/m^2^; and obese, ≥ 30 kg/m^2^ according to the WHO classification [19].

BMI binary (non-obese- BMI ≤ 29.9 kg/m2; obese- BMI ≥ 30 kg/m2) was considered for the purpose of logistic regression analysis.

### Covariates

Information on sociodemographic (sex, date of birth, years of education and region were collected at the baseline assessment (EpiDoC 1 study) and assumed constant throughout follow-up. Income (monthly wage/person) was also collected at baseline. The remaining measures were collected at the three waves (EpiDoC 1,2 and 3). In order to avoid possible confounders, we included as covariates: sex (male; female), age (years), participant residence according to the Portuguese Nomenclature of Territorial Units for Statistics II (NUTSII) (Norte, Centro, Lisboa e Vale do Tejo, Alentejo, Algarve, Açores e Madeira) [17], education level (years), income (monthly wage/person), type of work, obtained from job descriptions and classified into of two categories: white-collar profession (management and administrative jobs) and blue-collar profession (manual jobs), self-reported chronic disease (mental disease, rheumatic disease, cancer, allergy, pulmonary, gastrointestinal and urinary disease) as a binary variable response (yes; no), frequency of alcohol intake (daily; occasionally; never), smoking habits (daily; occasionally; past smoker; never smoked) and physical activity (yes; no).

### Statistical analysis

For the study population (in each wave and all waves together), sociodemographic characteristics, lifestyle behaviors and self-reported non-communicable chronic diseases were reported. Categorical variables were reported as absolute frequencies and percentages, and continuous variables as means and standard deviations.

Absenteeism was considered in two different ways: binary (absent from work: yes/no) and continuous (number of days absent from work).

To assess the association between absenteeism (yes/no) and obesity (obese/non obese), logistic regression was performed. The interactions sex*obesity and obesity*type of worker were tested but were not statistically significant. The models with and without interaction were compared and no statistically significant differences were found, which lead us to choose the most parsimonious model (without interaction). Therefore, three models were considered: Model 1 unadjusted model; Model 2 adjusted for sex and Model 3 adjusted for sex and type of worker (blue collar-white collar).

To determine the association between absenteeism (number of days) and obesity (BMI: underweight, normal weight, overweight and obese) a negative binomial mixed-effect model (NBMM) was used. Different statistical models were tested and based on the data’s distribution, model assumptions, fit and whether the model converged or not, NBMM was chosen. The choice of variables to include in the model was based on the previous use of these variables in similar analyses or their theoretical relevance for the association between obesity and absenteeism. Furthermore, the role of each potential covariate on the causal pathway between obesity and absenteeism and outcome and their biological relevance was carefully considered. The choice of potential variables to include was also limited but what was available on our dataset. The variables include were sex, age, NUTSII, education level, chronic non-communicable diseases (high blood pressure, high cholesterol levels, pulmonary disease, cardiac disease, gastrointestinal disease, mental disease, allergy, cancer, hyperuricemia, neurologic disease, urinary and rheumatic disease), lifestyles (alcohol, smoking habits and physical activity).

After selecting the potential confounders, different adjustments were tested: Model 1- adjusted for sex, age, NUTSII, education and time (fixed-effects); Model 2- adjusted for sex, age, NUTSII, education, time and chronic diseases (fixed-effects); Model 3- adjusted for sex, age, NUTSII, education, time and life-styles (fixed-effects); Model 4- adjusted for sex, age, NUTSII, education, time, chronic diseases and life-styles (fixed-effects). In all models the random effect considered was id. Time was only used as fixed effect since models failed to converge if different slopes per participant were included.

All analyses were performed on STATA v.15, and *p* < 0.05 was considered statistically significant.

### Obesity- attributable costs calculation

The costs of work absenteeism were estimated using the human capital approach (HCA), a method that estimates indirect costs due to productivity loss. The HCA considers the entire period of absence from work due to illness and values are based on achievable gross income [20].

Since EpiDoC only has family income and not individual, to calculate the total amount (€) lost in productivity, the average wages for each wave (EpiDoC1, EpiDoC 2 and EpiDoC 3) was calculated, by sex and age group, based on data available from the National Institute of Statistics (INE) [21]. In order to adjust these wages for the corresponding inflation period, an Actualization Factor (AF) was used based on rates of variation of the Consumer Price Index (CPI) from the INE, for the period of interest. The income value for each sex and group age was then multiplied by the respective AF to obtain each individual’s final estimated wage value.

Monthly wages were converted into yearly wages and divided by 253 to obtain the number of days worked and paid per year. For each individual, the yearly wages were multiplied by the number of missed workdays per person per year, to obtain the daily amount in euros spent for each day absent from work per individual/year. The prevalence of obesity was multiplied by the total number of active people employed in 2015, based on INE data, and the cost of absenteeism due to obesity per person/year was estimated for the entire population using the HCA.

## Results

### Sample characteristics at baseline

Of the 4338 active workers at baseline, most are women (57.2%), with a mean age of 42.7 ± 10.9 years and from the North, Center and Lisbon region (71.9%). Most participants have 10 or more years of schooling completed (53.4%). Most active workers are overweight (37%) or obese (15.2%)), with an average BMI of 25.9 ± 4.6 kg/m^2^. At baseline, 23.1% of participants reported missing work. Blue collar workers (61.9%) are more represented than white collar workers (38.1%) in our population (Table 1).

**Table 1:** Distribution of Sociodemographic and Employment Characteristics of Active Workers of the Epidemiological Study of the Cohort of Chronic Diseases (EpiDoc) 2011- 2016

**Variables**	**EpiDoC 1 (2011–13)**	**Missings**	**EpiDoC 2 (20,131–15)**	**Missings**	**EpiDoC 3 (2015–163)**	**Missings**	**All Waves (2011–16)**	**Missings**
	(*n* = 4338)	n (%)	(*n* = 3391)	n (%)	(*n* = 2444)	n (%)	(*n* = 10,173)	n (%)
**Sex, n (%)**		0		0		0		0
Male	1856 (42.8)		1402 (41.3)		1003 (41.0)		2292 (41.4)	
Female	2482 (57.2)		1989 (58.7)		1441 (59.0)		3246 (58.6)	
**Age, years, n (%)**		0		0		0		0
18–24	213 (4.9)		178 (5.2)		1003 (41.0)		470 (4.6)	
25–34	835 (19.2)		537 (15.8)		1441 (59.0)		1720 (16.9)	
35–44	1386 (32.0)		1029 (30.3)		79 (3.2)		3122 (30.7)	
45–54	1243 (28.7)		1067 (31.5)		348 (14.2)		3128 (30.8)	
55–64	606 (14.0)		518 (15.3)		707 (28.9)		1568 (15.4)	
≥ 65	55 (1.3)		62 (1.8)		818 (33.5)		165 (1.6)	
**Ethnicity, n (%)**		4 (0.2)		8 (0.2)		5 (0.2)		22 (0.2)
Caucasian/ White	4190 (96.8)		3291 (97.3)		444 (18.2)		9860 (97.1)	
Black	109 (2.5)		75 (2.2)		48 (2.0)		235 (2.3)	
Asian	4 (0.1)		2 (0.1)		2379 (97.3)		6 (0.1)	
Romany	2 (0.1)		2 (0.1)		51 (2.1)		5 **(**0.1**)**	
Other	24 (0.6)		13 (0.4)		-		45 (0.4)	
**Marital status, n (%)**		4 (0.2)		2 (0.1)		1 (0.0)		7 (0.1)
Single	872 (20.1)		773 (22.8)		8 (0.3)		2208 (21.7)	
Married	2666 (61.5)		2052 (60.5)		5 (0.2)		6214 (61.1)	
Divorced	424 (9.8)		309 (9.1)		563 (23.0)		938 (9.2)	
Widow(er)	124 (2.9)		82 (2.4)		1496 (61.2)		266 (2.6)	
Consensual	248 (5.7)		173 (5.1)		205 (8.4)		540 (5.3)	
**NUTS II, n (%)**		0		0		0		0
Norte	1233 (28.4)		997 (29.4)		60 (2.5)		2957 (29.1)	
Centro	724 (16.7)		641 (18.9)		119 (4.9)		1795 (17.6)	
Lisboa	1163 (26.8)		798 (23.5)		727 (29.7)		2525 (24.8)	
Alentejo	258 (5.9)		170 (5.0)		430 (17.6)		549 (5.4)	
Algarve	135 (3.1)		107 (3.2)		564 (23.1)		310 (3.1)	
Azores	417 (9.6)		352 (10.4)		121 (5.0)		1058 (10.4)	
Madeira	408 (9.4)		326 (9.6)		68 (2.8)		979 (9.6)	
**Educational level, years, n(%)**		14 (0.3)		10 (0.3)		6 (0.2)		30 (0.3)
0- 4	886 (20.5)		665 (19.7)		439 (18.0)		1990 (19.6)	
5 – 9	1130 (26.1)		847 (25.1)		562 (23.1)		2539 (25.0)	
10 – 12	1159 (26.8)		917 (27.1)		689 (28.3)		2765 (27.3)	
> 12	1149 (26.6)		952 (28.2)		748 (30.7)		2849 (28.1)	
**BMI (Kg/m**^**2**^**), n (%)**		72 (1.7)		182 (5.4)		72 (2.9)		326 (3.2)
Underweight (≤ 18.4)	66 (1.5)		61 (1.5)		46 (1.9)		173 (1.8)	
Normal (18.5–24.9)	1974 (46.3)		1482 (46.2)		1100 (46.4)		4556 (46.3)	
Overweight (25–29.9)	1577 (37.0)		1277 (38.2)		910 (38.4)		3714 (37.7)	
Obese (≥ 30)	649 (15.2)		439 (13.7)		316 (13.3)		1404 (14.3)	
**Absenteeism, n (%)**		132 (3.0)		257 (7.6)		589 (24.2)		852(8.4)
Yes	973 (23.1)		811 (25.9)		492 (26.6)		2262 (24.3)	
No	3233 (76.9)		2323 (74.1)		1357 (73.4)		7059 (75.7)	
**Type of Worker, n (%)**		2 (0.05)		14 (0.4)		41 (1.7)		57 (0.6)
White Collar	2683 (61.9)		2173 (64.3)		1598 (66.5)		6454 (63.8)	
Blue Collar	1653 (38.1)		1204 (35.7)		805 (33.5)		3662 (36.2)	
**Income, €, mean (± sd)**	1126.4 (194.1)		1097.4 (193.5)		1111.85 (182.0)		1113.27 (191.5)	
**Income, €, n (%)**		0		0		0		0
≤ 750	**-**		90 (2.7)		36 (1.5)		126 (1.2)	
751 to 1000	1390 (32.0)		1542 (45.5)		638 (26.1)		3570 (31.5)	
1001 to 1500	2920 (67.3)		1732 (51.1)		1753 (71.7)		6405 (63.0)	
≥ 1500	28 (0.6)		27 (0.8)		17 (0.7)		72 (0.7)	

Values are displayed as n (%) or mean (± sd). *NUTS II* Nomenclature of territorial units for statistics II, *BMI* Body Max Index

The most frequent comorbidities are high cholesterol (20.9%) and allergies (20.7%). Regarding lifestyles, most have never smoked (72.2%) and occasionally drink (47.3%). Only 38.1% of active workers practice exercise regularly (Table 2).

**Table 2:** Prevalence and 95% of Confidence Interval of Reported Chronic Disease and Lifestyle Habits of Active Workers of the EpiDoC Cohort 2011–2016

	**EpiDoC 1 (2011–13)**		**EpiDoC 2 (2013–15)**		**EpiDoC 3 (2015–16)**		**All Waves (2011–16)**	
	*n* = 4338		*n* = 3391		*n* = 2444		*n* = 10,173	
	***n (%)***	**95%CI**	***n (%)***	**95%CI**	***n (%)***	**95%CI**	***n (%)***	**95%CI**
***Chronic Disease***								
High blood pressure	704 (16.3)	(15.2–17.4)	548 (16.4)	(15.1–17.7)	411 (29.9)	(27.5–32.4)	1663 (18.4)	(17.6–19.2)
Diabetes Mellitus	187 (4.3)	(3.7–5.0)	142 (4.3)	(3.6–5.0)	106 (7.7)	(6.3–9.2)	435 (4.8)	(4.4–5.3)
High Cholesterol Level	896 (20.9)	(19.7–22.1)	742 (22.2)	(20.8–23.7)	484 (35.5)	(33.0–38.1)	2122 (23.4)	(22.7–24.5)
Lung Disease	133 (3.1)	(2.6–3.6)	112 (3.4)	(2.8–4.0)	37 (2.7)	(1.9–3.7)	282 (3.1)	(2.8–3.5)
Cardiac Disease	181 (4.2)	(3.6–4.8)	167 (5.0)	(4.3–5.8)	97 (7.0)	(5.7–8.5)	445 (4.9)	(4.5–5.4)
Gastrointestinal Disease	454 (10.5)	(9.6–11.5)	389 (11.6)	(10.5–12.7)	146 (10.6)	(9.0–12.3)	989 (10.9)	(10.3–11.6)
Neurological Disease	66 (1.5)	(1.2–1.9)	50 (1.5)	(1.1–2.0)	84 (6.1)	(4.9–7.5)	200 (2.2)	(1.9–2.5)
Allergies	893 (20.7)	(19.5–21.9)	723 (21.7)	(20.3–23.1)	293 (21.3)	(19.1–23.5)	1909 (21.1)	(20.3–22.0)
Mental Disease	462 (10.7)	(9.8–11.6)	387 (11.5)	(10.5–12.7)	335 (24.2)	(22.0–26.6)	1184 (13.1)	(12.4–13.8)
Cancer	82 (1.9)	(1.5–2.4)	77 (2.3)	(1.8–2.9)	72 (5.2)	(4.1–6.5)	231 (2.6)	(2.2–2.9)
Hyperuricemia	139 (3.2)	(2.7–3.8)	106 (3.2)	(2.6–3.8)	21 (1.5)	(0.9–2.3)	266 (3.0)	(2.6–3.3)
Renal Colic	251 (5.8)	(5.1–6.6)	203 (6.1)	(5.3–6.9)	47 (3.5)	(2.5–4.6)	501 (5.6)	(5.1–6.0)
Rheumatic Disease	609 (14.4)	(13.3–15.4)	649 (19.6)	(18.2–21.0)	539 (22.0)	(20.4–30.7)	1426 (14.3)	(13.6–15.0)
Obese	649 (15.3)	(14.2–16.4)	439 (13.1)	(12.0–14.3)	316 (13.3)	(12.0–14.8)	1404 (14.1)	(13.4–14.8)
***Lifestyle habits***								
**Alcohol**								
Never	1548 (35.8)	(34.4–37.2)	1125 (34.5)	(32.9–36.2)	628 (26.0)	(24.3–27.8)	3301 (33.0)	(32.1–33.9)
Occasionally	2048 (47.3)	(45.8–48.7)	1461 (44.7)	(43.2–46.6)	1137 (47.0)	(45.1–49.0)	4646 (46.5)	(45.5–47.4)
Daily	731 (16.9)	(15.8–18.0)	671 (20.6)	(19.2–22.0)	652 (27.0)	(25.2- 28.8)	2054 (20.5)	(19.8–21.3)
**Smoking habits**								
Never	3131 (72.2)	(70.9–73.5)	1753 (53.8)	(52.1–55.5)	1380 (57.0)	(55.0–59.0)	6264 (62.6)	(61.6–63.5)
Past smoking ^a^	Not applicable le	Not applicable	683 (21.0)	(19.6–22.4)	482 (19.9)	(18.4–21.5)	1442 (14.4)	(13.7–15.1)
Present smoker/occasionally	1204 (27.8)	(26.5–29.1)	821 (25.2)	(23.7–26.7)	559 (23.1)	(21.5–24.8)	2307 (23.0)	(22.2–23.9)
**Physical activity**								
Yes	1658 (38.1)	(36.7–39.6)	1700 (50.3)	(48.0–51.4)	1358 (44.4)	(42.5–46.4)	4439 (43.7)	(43.8–46.5)
No	2680 (61.9)	(60.4–63.3)	1679 (49.7)	(48.6–52.0)	1086 (55.6)	(53.6–57.5)	5717 (56.3)	(55.3–57.3)

^a^ Past smoker was not included at baseline

### Association between BMI and absenteeism

**Fig. 2:**
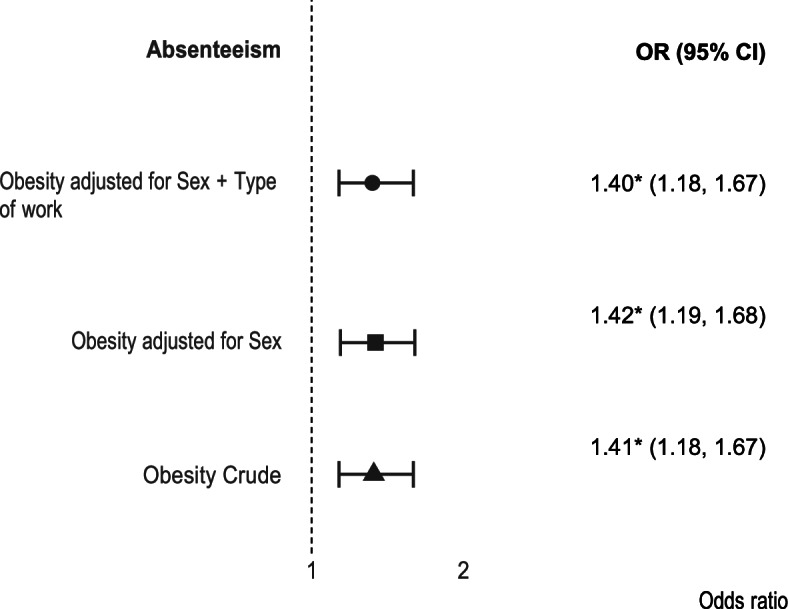
Forest plot of crude and adjusted odds ratios (with 95% confidence intervals) from logistic regression analysis. Statistically significant result (*)

First, the association between absenteeism and obesity was evaluated and the results are shown in Fig. 2. Obese had 40% higher odds of absenteeism compared with non-obese adjusted for sex and type of work (OR: 1.40; CI: 1.18–1.67, *p* < 0.01).

After evaluating the association between absenteeism (yes; no) and obesity, the association between the number of days absent from work and obesity was also assessed (Table 3).

**Table 3:** Association between days missed at work and Body Mass Index

	Mixed Effect Negative Binomial Regression			
	**Model 1 (*n* = 8991)**	**Model 2 (*n* = 6932)**	**Model 3 (*n* = 8956)**	**Model 4 (*n* = 7906)**
	(BMI)	(BMI + Chronic Diseases)	(BMI + Lifestyle Habits)	(BMI + Chronic Diseases + Lifestyle Habits)
	IRR (*p*-value)	IRR (*p*-value)	IRR (*p*-value)	IRR (*p*-value)
**BMI**				
Normal Weight	1	1	1	1
	-	-	-	-
Underweight	1.18 (0.685) [0.52–2.67]	1.33 (0.546) [0.53–3.35]	1.13 (0.771) [0.50–2.55]	1.34 (0.536) [0.53–3.37]
Overweight	1.14 (0.294) [0.89–1.46]	0.97 (0.925) [0.75–1.30]	1.14 (0.296) [0.89–1.46]	1.01 (0.968) [0.76–1.33]
Obese	**1.76 (0.001)** **[1.26–2.46]**	**1.63 (0.012)** **[1.12–2.38]**	**1.70 (0.002)** **[1.21–2.39]**	**1.66 (0.009)** **[1.13–2.44]**
**Time (years)**	1.11 (0.005) [1.03–1.20]	1.18 (0.003) [1.06–1.32]	1.11 (0.013) [1.02–1.20]	1.14 (0.037) [1.001–1.28]
**Gender**				
Male	1 -	1 -	1 -	1 -
Female	2.83 (< 0.001) [2.24–3.56]	1.88 (< 0.001) [1.44–2.44]	2.45 (< 0.001) [1.90–3.16]	1.79 (< 0.001) [1.35–2.38]
**Age group**				
18–24 years	1 -	1 -	1 -	1 -
25–34 years	2.24 (0.005) [1.27–3.94]	2.01 (0.024) [1.09–3.68]	2.32 (0.004) [1.32–4.08]	2.05 (0.021) [1.12–3.76]
35–44 years	2.00 (0.013) [1.16–3.46]	1.58 (0.123) [0.88–2.83]	2.09 (0.008) [1.21–3.62]	1.60 (0.116) [0.89–2.89]
45–54 years	2.07 (0.011) [1.18–3.60]	1.37 (0.303) [0.75–2.50]	2.20 (0.006) [1.26–3.53]	1.40 (0.274) [0.76–2.58]
55–64 years	1.79 (0.059) [0.98–3.29]	0.98 (0.953) [0.50–1.92]	1.91 (0.038) [1.04–3.53]	0.98 (0.962) [0.50–1.94]
65 or more years	1.64 (0.340) [0.59–4.57]	1.06 (0.921) [0.32–3.53]	1.61 (0.363) [0.58–4.49]	0.98 (0.969) [0.29–3.24]
**NUTS II**				
Lisboa	1 -	1 -	1 -	1 -
Porto	0.88 (0.421) [0.64–1.20]	0.87 (0.447) [0.62–1.24]	0.94 (0.715) [0.66–1.24]	0.88 (0.460) [0.62–1.24]
Centro	1.02 (0.922) [0.72–1.44]	1.04 (0.823) [0.71–1.54]	1.02 (0.931) [0.72–1.44]	1.01 (0.952) [0.69–1.49]
Alentejo	0.56 (0.038) [0.32–0.97]	0.63 (0.144) [0.34–1.17]	0.55 (0.034) [0.32–0.96]	0.59 (0.089) [0.32–1.08]
Algarve	1.01 (0.982) [0.52–1.95]	1.10 (0.806) [0.52–2.34]	1.02 (0.947) [0.53–1.99]	1.08 (0.837) [0.51–2.31]
Azores	0.57 (0.008) [0.37–0.86]	0.60 (0.035) [0.38–0.97]	0.58 (0.011) [0.38–0.88]	0.59 (0.029) [0.37–0.95]
Madeira	0.54 (0.007) [0.35–0.84]	0.52 (0.010) [0.32–0.86]	0.57 (0.012) [0.36–0.88]	0.51 (0.008) [0.31–0.84]
**Education level (years)**				
9 or less years	1 -	1 -	1 -	1 -
10–12 years	2.17 (< 0.001) [1.64–2.85]	2.08 (< 0.001) [1.44–3.00]	2.02 (< 0.001) [1.82–3.51]	1.99 (< 0.001) [1.46–2.71]
13 or more years	2.67 (< 0.001) [1.93–3.70]	2.10 (< 0.001) [1.54–2.85]	2.53 (< 0.001) [1.82–3.51]	2.06 (< 0.001) [1.41–2.99]
**Chronic Diseases**				
Mental	-	2.63 (< 0.001) [1.75–3.94]	-	2.49 (< 0.001) [1.66–3.75]
Rheumatic	-	2.94 (< 0.001) [2.07–4.18]	-	2.98 (< 0.001) [2.10–4.24]
Cancer	-	6.58 (< 0.001) [2.81–15.38]	-	6.43 (< 0.001) [2.76–14.98]
Allergies	-	1.35 (0.057) [0.99–1.84]	-	1.33 (0.074) [0.97–1.81]
Gastrointestinal	-	1.67 (0.013) [1.11–2.52]	-	1.69 (0.012) [1.12–2.54]
Lung disease	-	2.86 (0.003) [1.44–5.67]	-	2.85 (0.003) [1.43–5.64]
Renal Colic	-	2.57 (< 0.001) [1.52–4.36]	-	2.56 (< 0.001) [1.52–4.34]
**Alcohol**				
Never	-	-	1 -	1 -
Daily	-	-	0.50 (< 0.001) [0.35–0.70]	0.63 (0.026) [0.42–0.95]
Occasionally	-	-	0.68 (0.003) [0.53–0.87]	0.81 (0.137) [0.61–1.07]
**Smoking habits**				
Never	-	-	1 -	1 -
Daily	-	-	1.33 (0.042) [1.01–1.75]	1.34 (0.061) [0.99–1.82]
Occasionally	-	-	0.55 (0.078) [0.28–1.07]	0.77 (0.486) [0.36–1.62]
In the past	-	-	1.61 (0.011) [1.12–2.33]	2.01 (0.003) [1.26–3.21]
**Regular Exercise**				
No	-	-	1 -	1 -
Yes	-	-	0.74 (0.008) [0.60–0.92]	0.79 (0.070) [1.26–3.21]

*Notes:*

1) All regressions were adjusted to age, sex, education level, NUTII and time

2) Chronic Disease means all diseases with p value ≤ 0.02 level of significance on the hypothesis tests, including mental disorder, rheumatic disease, cancer, gastrointestinal disease, allergies, lung disease, renal colic. 3) Lifestyle habits include alcohol, tobacco, and physical activity

*IRR* Incidence rate ratio

*CI* Confidence interval

*BMI* Body Mass Index

Normal Weight – 18.5 -24.9 kg/m2; BMI Overweight 25.0.29.9 kg/m2; BMI; Obese >  = 30.0 kg/m2 BMI

NUTT—Nomenclature of Territorial Units for Statistics

Obesity was positively and significantly associated with absenteeism for all adjustments considered.

Considered the simplest model, adjusted for sex, age, education level and NUTII, obese workers missed 76% more days at work than workers with normal weight during the study period (IRR: 1.39, 95%CI: [1.26–2.46]; *p* = 0.001).”When we consider the more complex model, adjusted for sex, age, education level, NUTII, chronic diseases and lifestyles, workers missed 66% more days at work than workers with normal weight during the study period (IRR: 1.66, 95%CI: [1.13–2.44]; *p* = 0.009 (Table 3).

### Differences in absenteeism days and indirect costs across BMI categories

When assessing the total number of workdays missed by normal weight and obese employees, we found that obese employees miss work on average 10.2 days per year, which is one average 3.8 more workdays per year than normal-weight employees.

In men, obese individuals miss working one average 3.5 more days per year than men’s with normal weight. In women employees who were obese missed more 4.6 workdays on average per year than those with normal weight.

The indirect cost of absenteeism due to obesity for the total population was calculated, and we found that this ranged from €391.1 to €426.52 per employee per year (Table 4). In addition, for obese individuals, absenteeism cost an average of €160.4 more than for individuals with normal weight. For each obese adult man, we estimated the cost of obesity to be €521.5 per year, ranging from €307.7 to €735.3, and for each obese woman it was €575.0 per year, ranging from €434.2 to €715.8.

**Table 4:** Average days missed at work per year and its cost according to gender and body mass index

	**Average annual number of workdays absent due to health (95% CI)**	**Additional absent workdays compared to normal weight (95% CI)**	**Cost of absenteeism per employee/year (95% IC)**	**Additional cost of absenteeism per employee/year (95% IC) compared to normal weight (95% CI)**	**Cost of absenteeism due to obesity /year for Portugal**	**Additional cost of absenteeism due to obesity/year for Portugal compared to normal weight**
**Overall**	7.3 (6.7–8.1)		391.1 € (355.6 € 426.5€)			
Normal weight	6.4 (5.2–7.5)		432.9.0 € (368.9.4€497.3€)			
Overweight	8.2 (7.0—9.4)	1.8 (1.1–2.5)	432.9.0€ (368.9.4€497.3€)	41.8 € (6.78€- 89.4 €)		
Obese	10.2 (8- 12.4)	3.8 (3.1–4.5)	551.5€ (429.0€- 674.0€)	160.4€ (119.0€-201.7€)	819 433,70 €	238 326,69 €
**Men**						
Normal weight	4.5 (3.4–5.6)		271.4€ (1201.3.5341.4€)			
Overweight	5.1 (3.8–6.3)	0.5 (-0.5–1.5)	325.3€ (245.19€- 405.40€)	53.9 € (10.95€-118.7 €)		
Obese	8 (4.7 -11.2)	3.5 (2.46–4.5)	521.5€ (307.7€- 735.3€)	130.4 € (65.5€-195.2 €)	774 858,889 €	341 582,10 €
**Woman**						
Normal weight	7.4 (6.2–8.6)		351.3 € (294.7€- 408.0€)			
Overweight	11.4 (9.2–13.5)	4.0 (2.8–5.1)	543.6 € 442.45€- 644.97€)	192.3 € (138.7€-245.8 €)		
Obese	12 (11.2–12.8)	4.6 (3.4–5.7)	575.0 € (434.2€-715.8€)	223.7 € (170.1€-277.26€)	854 350,64 €	332 349,41 €

Compared to normal weight and adjusted to age, sex, education level, NUTII, chronic disease and lifestyle habits

Normal Weight – 18.5 -24.9 kg/m2; BMI Overweight 25.0-29.9 kg/m2; BMI; Obese >  = 30.0 kg/m2 BMI

Overall, the indirect cost of absenteeism per year for obese employees in Portugal was predicted at €819million, which is on average €238 million more per year for obese people when compared with people with normal weight (Table 4).

## Discussion

In this study, we investigated the association between obesity and absenteeism. obese workers missed 66% more days at work than workers with normal weight, independent of other risk factors, including chronic disease and unhealthy lifestyle choices.

We estimate that absenteeism due to obesity imposes a considerable financial burden on states, totaling an additional €238million per year in Portugal.

In a previously published study, obesity was found to increase the absenteeism incidence rate ratio by approximately 27%, than normal weight peers [22]. Some authors, when applying a longitudinal methodology to understand the causal relationship between obesity and absenteeism, found that obesity could act both as a direct explanatory variable and as a mediator for other variables linked to loss of productivity, as obesity is also considered a risk factor for several chronic diseases [22, 23]. BMI also, appears to exert a causal effect on employment status, largely by affecting an individual’s health rather than through increased unemployment arising from social discrimination [24]. These results are consistent with our findings showing similar degrees of obesity-associated effects on absenteeism, both when it is evaluated separately and when associated with other risk factors, such as chronic diseases and lifestyle habits. However, as has been recommended by other authors, prospective analyses are necessary to determine the time of occurrence, that is, whether diseases occur before or after an individual has become overweight or obese. Such studies would help to establish a clear causal framework for a meaningful attribution of the indirect costs of obesity [23].

Obese workers are more likely to report poor work ability or limitations in the amount, type, or quality of work, than their normal-weight counterparts [25]. In the present study, we found that obese workers were 40% (*P* < 0.01) more likely to miss work than normal weight peers. Furthermore, when we tested the interactions between obesity and sex, and obesity and type of professions, these were not statistically significant. This result is not aligned with a study from the United States, for example, that found the probability of missing work was significant across all professional categories for obese women, although among men, the results varied by occupation [26]. This discrepancy with our findings can by justified due to a much more complex mechanism, including differences in the democracy, as well in the economic situation, labour market and social welfare between Portugal and United States that could be considered as potentially important factor that explaining differences in population health behaviors and consequently in absenteeism [27, 28].

Numerous studies have shown that obesity is strongly associated with absenteeism. Finkelstein et al*.* reported that grade-I obese women (BMI, 30–34.9) in the United States miss 5.2 days per year due to illness or injury, which is 1.8 days more than normal-weight women, while grade-II (BMI, 35–39.9) and grade-III (BMI, ≥ 40) obese women miss 3.0 and 4.8 more days, respectively, than normal-weight women, with all increases statically significant [29]. In contrast, grade-II and grade-III obese men miss approximately two more workdays per year than normal weight men, similar to what has been found in other studies [26, 29].

In our study, we found that obese individuals miss 10.2 workdays on average per year, 3.8 more than their normal-weight peers. Obese women and men lose 12 and 8 working days per year, respectively, which is 4.6 and 3.5 days more, respectively, than their normal weight counterparts. Similarly, a study conducted in London reported a loss of 9.5 workdays per year for obese workers. European countries have many common characteristics, relating to population distribution, sociodemographic characteristics, and prevalence of obesity itself. Thus, it makes sense that our results are more similar to those reported in London [30] than to findings from the United States [25, 26, 29]. Regardless, all these studies report the key finding that obesity has a substantial impact on lost working days [26, 30]. Adiposity and fat distribution are closely associated with whole body metabolism and long-term health, and consequently, obese individuals often have more chronic conditions associated with poor health status. Thus, we expect that this is the reason that people with obesity display greater absence from work due to sickness, as well as increased healthcare consumption, relative to their non-obese counterparts.

Critically, absenteeism due to obesity imposes a considerable financial burden on states. In this study, we estimated that obese workers incur an additional cost of €238 million per year when compared to non-obese workers. These costs range from to €551 to €674 per year for each obese employee and are higher for women than men (€575.0 *vs*. €521.5, respectively). A previous study by Pereira et al*.* estimated the total indirect cost of obesity in Portugal in 2002 at €199,8 million, indicating that obesity causes considerable economic losses for the country [27]. Comparison with our findings reveals a difference of €37 million between the study by Pereira et al*.* and our estimate for obesity-associated costs due to absenteeism per year. Despite that, it is important to say that the methodology between the two studies is different, while Pereira et al.used Attributable Risk to calculate the number of deaths attributable to obesity, taking into account the relative risk of obesity prevalence estimates, [31], in our study we used absenteeism as the main outcome and estimated the costs through the HCA extrapolating to all population using the prevalence of obesity in the years of study.

A recent international literature review, reported obesity-attributable productivity losses ranging from $89 to $1586 for absenteeism [23], while in in Europe obese individual range €1031 to €1700 whereas individuals with BMI ≥ 40 kg/m2 reported almost €2,000 more indirect costs annually compared with normal weight respondents [32].Our study showed that absenteeism costs in Portugal are in the lower end of the European spectrum (~ €551 in a spectrum of €1031to €1700). A possible explanation is that Portugal is one of the countries in Europe that offers its workers the lowest minimum wages, this is also a public health concern once there is corresponding effects of income on health, so if we compare the working day in Portugal with countries that have higher wages, probably the proportion of money lost for the same days off work, it will always be lower [33].

Further, international studies have attributed increases in health sector spending ranging from 30–60% to absenteeism, with indirect costs reaching $8.65 billion per year in 2012 [22, 29]. Thus, the economic impact of obesity on both the health sector and society as a whole is undeniable, and numerous studies have reported this issue as a critical problem that is increasing in prevalence. Therefore, we expect that the implementation of strategies to prevent obesity could generate gains in productivity, and reduce the economic burden imposed by this disease. In Portugal the National Program for the Promotion of Healthy Eating (PNPAS) despite being recent having started only in 2012, in a short period of time achieve some important results, as a the reduction of salt and sodium in bread, the taxation of sweetened beverages and change in the availability of unhealthy foods, particularly in school, work and in public spaces, what certainly contribute to prevent obesity in general population. However, the effectiveness of this policies on obesity reduction among working adults and their effect on absenteeism still remains to explore [34–36].

We note, however, that this study has several limitations. First, the BMI values were based on self-reported weight and height, which could generate systematic bias, as people often underreport their weights and overestimate their heights [37]. Second, our dependent variable, absenteeism, had a high number of zero observations, which substantially reduces the study’s sample size. However, by using appropriate statistical modelling and comparing different statistical approaches, we trust that our data are sufficiently robust to support our conclusions [38, 39]. We consider that EpiDoC dataset is sound because this cohort has a representative sample of Portuguese adults including the active workers. Data collection was made using structured questionnaires and performed by trained research assistants. A comprehensive set of variables in each wave of follow-up was collected from each participant with reduced missing data [17].

An additional challenge arises from the fact that, to the best of our knowledge, there is no standard methodology reported in the literature for evaluating the indirect cost of absenteeism due to obesity [8, 14, 40]. This study might underestimate the indirect costs of absenteeism since it only considers the number of absence days from work. The decreased health or disease might affect also other indirect costs which were not considered, such as presenteeism while at work, and reduced informal work. Further, it is important to note that the salary values in this study were estimated based on the imputation of average worker wage data corresponding to the evaluation period of the EpiDoC and may therefore not be completely accurate and due to this wage variable having originated from a secondary base, in which there was no classification of the population according to the type of profession, we chose not to stratify the means based on these subgroups. In addition, we assessed the cost of obesity with the HCA, which takes both an individual and societal perspective and has often been criticized for overestimating loss of productivity This overestimation may be due to the fact that the amount spent for each hour not worked in the long term is related to the early retirement age whereas the human capital method may estimate potential costs rather than actual costs [20].

Despite these limitations, our study also has a number of key strengths. As previously mentioned, our data were obtained from a cohort representing the entire adult Portuguese population, and repeated measures can evaluate changes in outcomes over time. We also evaluated costs using obesity as the main predictor variable, whereas most other studies evaluated costs related to obesity-related diseases, and not just obesity. For this reason, we expect that our study is less prone to confounding bias and thus, better able to determine associations between obesity and absenteeism and estimate its costs. Furthermore, our dataset containing information relating to days absent, chronic diseases, and lifestyle factors is quite robust for indirect cost estimates and allows for a comprehensive analysis of obesity-associated factors.

The use of objectively measured height and weight to derive BMI and classify individuals into obesity should be a priority for future studies. Furthermore, pending request and approval, the social security database, and the primary care centre database (where height and weight are routinely measured) could be fully or partly linked to provide a comprehensive and updated picture of the impact of obesity on absenteeism.

## Conclusion

In this study, we analyzed data from a large Portuguese population-based cohort. We found that obesity is an independent risk factor for absenteeism that imposes a severe economic burden on society. Critically, future studies aimed at evaluating the cost-effectiveness and efficacy of specific interventions in the workplace for preventing obesity could yield key insights on the best methods and most effective policies for addressing this public health crisis and reducing obesity associated costs.

## Supplementary Information

**Additional file 1.**

## Data Availability

Data that supported this article is available on Dias SS, Rodrigues AM, Gregório MJ, de Sousa RD, Branco JC, Canhão H. Cohort Profile: The Epidemiology of Chronic Diseases Cohort (EpiDoC). Int J Epidemiol. 2018;47(6):1741-2j. https://doi.org/10.1093/ije/dyy185

## Abbreviations

NUTS II: Nomenclature of Territorial Units for Statistics
BMI: Body Mass Index
EpiDoC: Epidemiology of Chronic Diseases Cohort
GDP: Gross Domestic Product
HCA: Human Capital Approach
INE: National Institute of Statistics
AF: Actualization Factor
CPI: Consumer Price Index
IRR: Incidence rate Ratio
OR: Odds Ratio
CI: Confidence Interval
