# Correction: Obesity- attributable costs of absenteeism among working adults in Portugal

**DOI:** 10.1186/s12889-022-13483-4

**Published:** 2022-06-17

**Authors:** Kelli Destri, Joana Alves, Maria João Gregório, Sara Simões Dias, Ana Rita Henriques, Nuno Mendonça, Helena Canhão, Ana Maria Rodrigues

**Affiliations:** 1 Comprehensive Health Research Centre, NOVA Medical School, Universidade Nova de Lisboa, 1169-056 Lisboa, Portugal; 2 EpiDoC Unit, CEDOC, NOVA Medical School, Universidade Nova de Lisboa, Lisboa, Portugal; 3 Comprehensive Health Research Center (CHRC), NOVA National School of Public Health, Public Health Research Centre, Universidade NOVA de Lisboa, Lisbon, Portugal; 4 Programa Nacional Para a Promoção da Alimentação Saudável, Direção-Geral da Saúde, Lisbon, Portugal; 5 Faculdade de Ciências da Nutrição E Alimentação, Universidade Do Porto, Porto, Portugal; 6 Center for Innovative Care and Health Technology, CiTechCare, Instituto Politécnico de Leiria/Escola Superior de Saúde, Leiria, Portugal; 7 National School of Public Health, UNL, Lisboa, Portugal; 8 Hospital Dos Lusíadas, Lisboa, Portugal

**Correction: BMC Public Health 22, 978 (2022)**

**https://doi.org/10.1186/s12889-022-13337-z**

In the original publication of this article an error was introduced during the publication process. Due to this error: Figures 1 and 2 were identical. The article [1] has been updated with the correct figure 1. The publisher apologizes for the inconvenience caused to the authors & readers.
