# Supplementary material for: Obesity- attributable costs of absenteeism among working adults in Portugal
Source: BMC Public Health. 2022 May 15;22:978. doi: 10.1186/s12889-022-13337-z (PMC9107744; doi:10.1186/s12889-022-13337-z)
Supplement: Supplementary file 1 — Additional file 1. [file 12889_2022_13337_MOESM1_ESM.docx]

###### Supplementary material


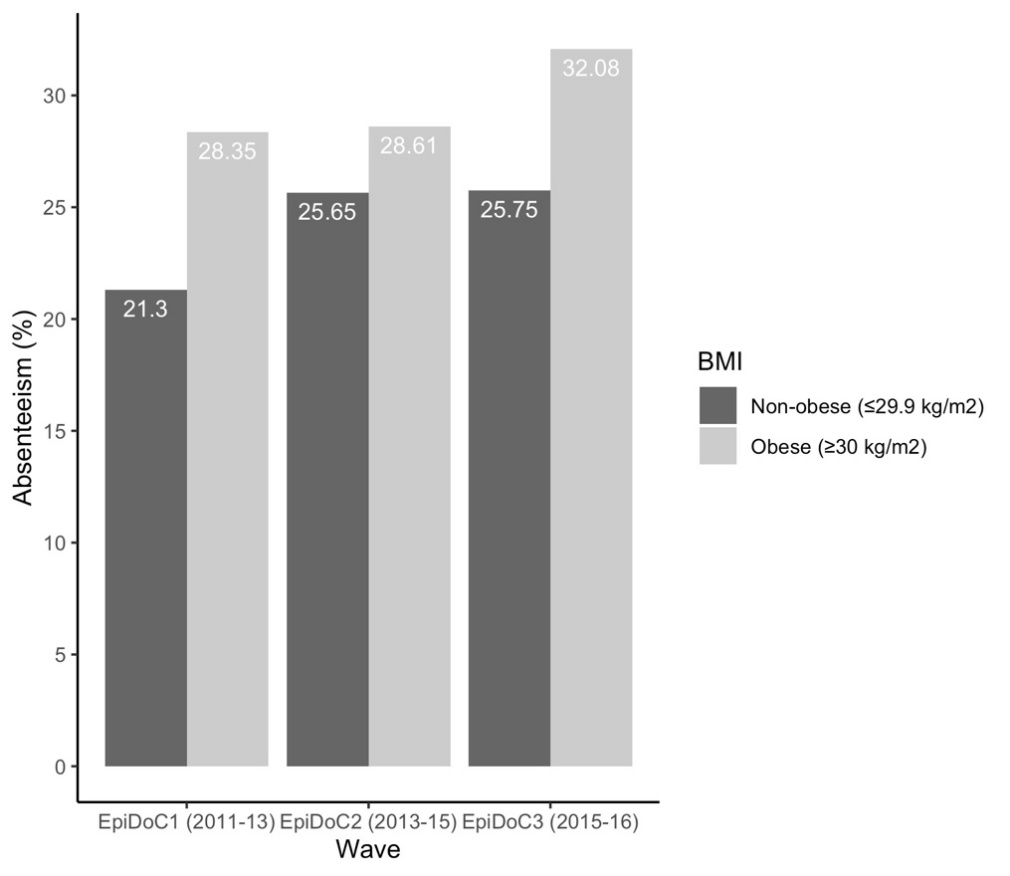


###### Figure S1- Prevalence of absenteeism in each time-point considered (Obese vs non-Obese)
